# Oral Health Implications of SARS-CoV-2/COVID-19: A Systematic Review

**DOI:** 10.3290/j.ohpd.b2960801

**Published:** 2022-04-27

**Authors:** Maria Vilar Doceda, Marianna Gavriiloglou, Catherine Petit, Olivier Huck

**Affiliations:** a Postgraduate Student, Université de Strasbourg, Faculté de Chirurgie Dentaire, Periodontology, Strasbourg, France; Hôpitaux Universitaires de Strasbourg, Pôle de médecine et chirurgie bucco-dentaires, Strasbourg, France. Searched the litterature, wrote the manuscript.; b Postgraduate Student, Hôpitaux Universitaires de Strasbourg, Pôle de médecine et chirurgie bucco-dentaires, Strasbourg, France; Université de Strasbourg, Faculté de Chirurgie Dentaire, Periodontology, Strasbourg, France. Searched the literature, wrote the manuscript.; c Associate Professor, Université de Strasbourg, Faculté de Chirurgie Dentaire, Periodontology, Strasbourg, France; Hôpitaux Universitaires de Strasbourg, Pôle de médecine et chirurgie bucco-dentaires, Strasbourg, France; INSERM (French National Institute of Health and Medical Research), UMR 1260, Regenerative Nanomedicine, Fédération de Médecine Translationnelle de Strasbourg (FMTS), Strasbourg, France. Critically revised the manuscript.; d Professor, Université de Strasbourg, Faculté de Chirurgie Dentaire, Periodontology, Strasbourg, France; Hôpitaux Universitaires de Strasbourg, Pôle de médecine et chirurgie bucco-dentaires, Strasbourg, France; INSERM (French National Institute of Health and Medical Research), UMR 1260, Regenerative Nanomedicine, Fédération de Médecine Translationnelle de Strasbourg (FMTS), Strasbourg, France. Designed the study protocol, critically revised the manuscript.

**Keywords:** COVID-19, oral health, oral manifestation, periodontal diseases, SARS-CoV-2

## Abstract

**Purposes:**

The aim of this sytematic review was to evaluate the potential association of COVID-19 infection with oral health.

**Materials and Methods:**

Screening in different databases (PubMed/MEDLINE, Google Scholar, and Embase databases) was performed to identify relevant articles, focusing on the oral health of patients with COVID-19, and published up to November 2021. 5194 articles were identified, and 29 fulfilled the inclusion criteria.

**Results:**

Patients presenting more severe periodontal or dental diseases were at an increased risk of developing COVID-19 complications and being admitted to intensive care units. According to the included articles, U-shaped lingual papillitis and aphthous-like ulcers on the tongue are the most frequent lesions assessed in the oral cavity of COVID-19 patients, while xerostomia seems to be an early COVID-19 diagnostic symptom. Apart from the presence of the virus, the global lockdown had a detrimental impact on oral health. The occurrence of dental emergencies was augmented during this time due to the postponement of numerous non-emergency dental procedures.

**Conclusions:**

The presence of SARS-CoV-2 in periodontal tissues and salivary fractions may explain the presence of oral lesions during the infection. However, the virus’s direct or indirect effect on oral mucosa is unclear. It is important to consider that these manifestations might be attributed to underlying comorbidities, or co-existing or subsequent lesions produced by local irritants.

Since the first cases diagnosed with an unidentified severe pneumonia in Wuhan, Hubei province, China, the disease named COVID-19 became a global matter. The high rate of spread led the World Health Organisation (WHO)^13^ to declare COVID-19 a pandemic.^59^ By December 2021, 268,934,575 cases and 5,297,850 deaths had been confirmed. In a recent meta-analysis, the case-fatality rate was estimated at around 1% among the general population and 19.0% in people over 50 years old.^2^

The new virus responsible for COVID-19 is a member of the Coronaviridae family and is called severe acute respiratory syndrome coronavirus 2 (SARS-CoV-2).^59^ A wide range of clinical features are known, from asymptomatic infection to severe disease and death.^49^ The most common symptoms are fever, cough, sore throat, headache, fatigue, muscle aches and dyspnea.^49^ Loss of olfaction and gustation have also been observed, with a recovery rate of more than a month in half the cases.^35^

SARS-CoV-2 has demonstrated a tissue tropism towards nasal, ocular, and oral tissues.^34^ Indeed, several studies demonstrated the presence of SARS-CoV-2 in the oral cavity and salivary secretion even in the asymptomatic population.^25^ This could be explained by the high expression in oral tissues of angiotensin-converting enzyme 2 (ACE2), which is the primary receptor for severe acute respiratory syndrome coronavirus.^61^ Oral tissues are prone to viral infection and viruses; for instance, herpesviruses, papillomaviruses, torque teno viruses and human immunodeficiency virus are commonly detected.^40^ Herpesviruses, for example, have the ability to generate latent infection and active replication in different cell types of the periodontium under environmental stress. Nevertheless, human cytomegalovirus (HCMV) and Epstein-Barr virus (EBV), both members of the herpesvirus family, have been suggested to play a role in the pathogenesis of periodontitis and symptomatic periapical lesions, either by inducing immunosuppression or by directly infecting periapical cells.^44^

Interestingly, clinical oral manifestations in patients with COVID-19^9^ and a rapid progression of periodontal tissue breakdown have been observed during the COVID-19 pandemic.^31^ We hypothesised that SARS-CoV-2 infection and COVID-19 are associated with worsened periodontal conditions and the onset of oral diseases.

The aim of this systematic review was to identify the most common signs and symptoms of SARS-CoV-2 in the oral cavity as well as the indirect impact of social restrictions and isolation periods in the context of the COVID-19 pandemic.

## Materials and Methods

### Focused Question

In this systematic review, the following question was addressed: Is there any association between oral health and COVID-19?

### Screening and Selections of Papers

The present systematic review complied with the PRISMA statement.^38^ A literature search was performed by two blinded researchers (MVD, MG) and confirmed by another (OH). Relevant studies were identified from: PubMed/MEDLINE, Google Scholar, and Embase databases, up to and including November 2021. The following search terms were included: “oral lesion” OR “oral manifestation” OR “periodontal disease” OR “periodontitis” AND “COVID-19” OR “SARS-CoV-2”. After screening the titles and abstracts of the publications, eligible studies were obtained. Discussion between authors was conducted in case of disagreement. Finally, all the identified articles were assessed individually.

### Type of Studies

Studies published in English and reporting at least one oral manifestation in COVID-19 patients were included. Review articles, letters to editors, brief communications, case reports and articles not in the English language were excluded. Studies which reported only gustatory and olfactory disorders (dysgeusia or ageusia) in patients positive for SARS-CoV-2 infection were also excluded.

### Outcomes

All oral health-related manifestations (periodontal disease, mucosal lesions, salivary changes, infection, etc) were considered in the analysis of the identified publications.

### Quality Assessment

A quality assessment of all included studies was performed according to Baccaglini et al^6^ by two independent reviewers (MVD, MG). Disagreements were discussed and solved by reaching a consensus. Quality of evidence was estimated using the following items: allocation concealment (selection bias), random sequence generation, blinding of personnel and participants (performance bias), incomplete outcome data (attrition bias), blinding of outcome assessment (detection bias), selective reporting (reporting bias), and other sources of bias (no description of findings, patient profile, time from diagnosis, or drug use) (Table 1).

**Table 1:** Quality of assessment of included studies

Study	Bias
Marouf et al 2021	B
Larvin et al 2020	C
Anand et al 2021	C
Iwasaka et al 2021	C
Fernades Matuck et al 2021	F
Gupta et al 2021	A
Politi et al 2020	C
Wu et al 2021	C
Ramírez et al 2021	C
Fantozzi et al 2020	C
Freni et al 2020	B
Biadsee et al 2020	C
Sirin et al 2021	C
Salehi et al 2020	C
Hocková et al 2021	B
Sinjari et al 2020	B
Favia et al 2021	C
Huang et al 2021	B
Jimenez-Cauhe et al 2020	C
González et al 2021	C
Fidan et al 2021	B
AbuBakr et al 2021	C
Subramaniam et al 2021	B
Emodi Perlman et al 2020	C
Asquini et al 2021	A
Petrescu et al 2020	B
Zhang et al 2021	C
Kumar et al 2021	B
Kamel et al 2021	C

CI: confidence intervals; OR: odds ratio; NR: not reported; NA: no answer; RT-PCR: reverse transcription polymerase chain reaction; ICU: intensive care unit; IV: intravenous; LA: local anaesthesia; GA: general anaesthesia; MIS-C: multisystem inflammatory syndrome in children; TMD: temporomandibular disorders; AB: awake bruxism; SB: sleep bruxism.

## Results

### Study Selection

**Fig 1:**
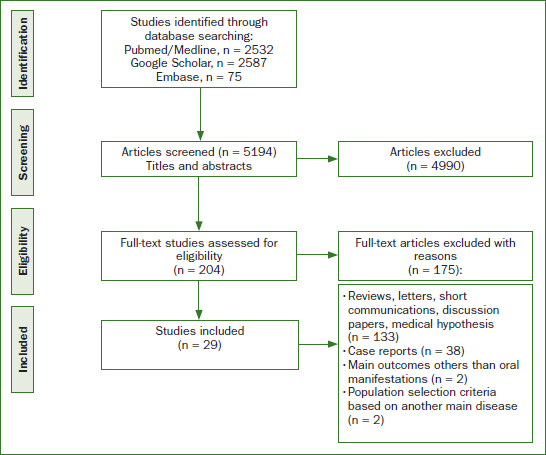
Flow diagram of literature search and inclusion

Seeking the correlation between the COVID-19 pandemic and oral changes, a total of 5194 studies were identified (Fig 1). After screening of titles and abstracts, 204 studies were included, and 4990 were found to be irrelevant. After full-text assessment, 175 studies were excluded due to the inclusion/exclusion criteria. Thus, 29 studies were finally included in this systematic review.

### Study Characteristics

The main characteristics of the included articles are summarised in Table 2. Among the 29 included studies, 5 were case series, 7 were retrospective and 5 were prospective cohort studies, 9 were cross-sectional studies, and 3 were case-control studies, published up to November 2021. The included studies were conducted in diverse countries across Asia, Europe, and America with participant group sizes ranging from 13,253 to 7. The listed studies evaluated different aspects of COVID-19–associated oral manifestations, including periodontal diseases, xerostomia, temporomandibular disorders, and bruxism, dental damage, or candidiasis. The selected studies used different methods (questionnaires, radiographs, clinical examinations, biopsies, and RNA sample analysis) to evaluate possible association.

**Table 2:** Characteristics of the included studies

Study	Design	Sample size and gender	Age in years Mean ± SD or range	COVID-19 severity, n	Oral signs and symptoms reported, n	Results
Marouf et al (2021)	Case control	n = 568 Control: n = 528 M = 290 (54.9%) F = 238 (45.1%) Case: n = 40 M = 20 (50%) F = 20 (50%)	Control = 41.5 ±14.1 Case = 53.6 ±15.0	Control: Covid positive patients without complications (n = 528) Case: Covid positive patients with complications (n = 40): Death = 14 ICU admission = 36 Need for assisted ventilation = 20	Periodontally healthy or initial periodontitis (stages 0-1): Bone loss < coronal third of the root length (15%) in OPGs, or ≤2 mm in bitewing radiographs. Control = 303 Case = 18 Periodontitis (stages 2-4): Bone loss > coronal third of the root length (>15%) in OPGs, or >2 mm in bitewing radiographs. Control = 225 Case = 92	Periodontitis associated with COVID-19 complications: Death (OR = 8.81, 95% CI 1.00-77.7) ICU admission (OR = 3.54, 95% CI 1.39-9.05) Need for assisted ventilation (OR = 4.57, 95% CI 1.19- 17.4)
Larvin et al (2020)	Case control	n = 13.253 Control: n = 9.815 M = 4789 (48.8%) F = 5026 (51.2%) Case: n = 1.338 M = 711 (53.1%) F = 627 (46.9%)	Control = 69.10±8.20 Case = 67.15 ±9.19	No self-reported history of periodontal disease: Control = covid negative patients = 9.815 Death = 292 (3.0%) Hospital admission = 2.723 (27.7%) Case = Covid positive patients = 1.338 Death = 247(18.5%) Hospital admission = 665 (49.7%)	Painful gums Control = 321 Case = 44 Bleeding gums Control = 1148 Case = 181 Loose teeth Control = 353 Case = 53	COVID-19 positive participants: Painful or bleeding gums had a higher risk of mortality (OR: 1.71, 95% CI: 1.05–2.72) but not hospital admission (OR: 0.90, 95% CI: 0.59–1.37). Loose teeth did not show higher risk of hospital admission or mortality compared to the control group (OR = 1.55, 95% CI: 0.87–2.77; OR: 1.85; 95% CI: 0.92–2.72)
Fernandes Matuck et al (2021)	Case series	n = 7 M = 3 (42.85%) F = 4 (57.14%)	47.4 (8-74)	Death = 7	Periodontal tissue positive for SARS-CoV-2 (RT-PCR) = 5 Periodontal tissue negative for SARS-CoV-2 (RT-PCR) = 2	Presence of SARS-CoV-2 in periodontal tissue in COVID-19 positive patients
Gupta et al (2021)	Cross-sectional study	n = 82 M = 48 (58.5%) F = 34 (41.46%)	Periodontally healthy = 34.44 ± 11.06 Gingivitis = 37.71 ± 10.0 Stage I periodontitis = 52.33 ± 16.25 Stage II periodontitis = 44.00 ± 18.38 Stage III periodontitis = 62.94 ± 12.76 Stage IV periodontitis = 61.58 ± 9.07	COVID-19 positive patients confirmed by nasopharyngeal swab (NPS) testing = 82 - COVID-19 symptoms: Symptomatic = 51 Asymptomatic = 31 - Hospital admission = 53 - Oxygen requirement = 30 - COVID-19 pneumonia = 22 - Deceased = 8	Periodontally healthy = 27 Gingivitis = 21 Stage I periodontitis = 3 Stage II periodontitis = 2 Stage III periodontitis = 17 Stage IV periodontitis = 12	Higher severity of periodontitis led to 7.45 odds of requiring assisted ventilation, 36.52 odds of hospital admission, 14.58 odds of being deceased and 4.42 odds of COVID-19-related pneumonia
Anand et al (2021)	Case control	n = 150 Control = 71 M = 35 F = 36 Case = 79 M = 50 F = 29	Control = 38.24 ± 10.72 Case = 43.34 ± 10.16	Control: Covid negative patients (n = 71) Case: Covid positive patients after rRT-PCR (n = 79)	Plaque score ≥ 1: - Control: 3 (4.2%) - Case: 19 (24.1%) Gingivitis: - Control: 36 (50.7%) - Case: 74 (93.7%) Mean CAL ≥ 2: - Control: 15 (21.1%) - Case: 51 (64.6%) Severe periodontitis - Control: 7 (9.9%) - Case: 39 (49.4%)	Periodontitis associated with COVID-19. Mean plaque scores ≥ 1 (OR, 7.01; 95% CI, 1.83-26.94) Gingivitis (OR, 17.65; 95% CI, 5.95-52.37) Mean CAL ≥ 2 mm (OR, 8.46; 95% CI, 3.47-20.63) Severe periodontitis (OR, 11.75; 95% CI, 3.89- 35.49)
Iwasaki et al (2021)	Cross-sectional questionnaire regarding the status of regular dental visits during the COVID-19 pandemic	n = 199 M = 123 (61.8%) F = 76 (38.2%)	42.6 ± 10.4	NR Japanese office workers during the COVID-19 pandemic	Group that continued regular visits (n = 77): - Periodontitis = 38 (49.4%) - Severe periodontitis = 4 (5.2%) Group that discontinued regular dental visits (n = 31) - Periodontitis = 24 (77.4%) - Severe periodontitis = 1 (3.2%) Group that did not attend regular dental visits (n = 91) - Periodontitis = 56 (61.5%) - Severe periodontitis = 10 (11.0%)	Individuals who discontinued regular dental visits had a higher prevalence of periodontitis (49.4% vs 77.4%, p < 0.05) and concerns regarding dental visits (22.1% vs 64.5%, p < 0.05). Discontinuing regular dental visits significantly mediated the association between concerns regarding dental visits and periodontitis (natural indirect effect: odds ratio = 1.68, 95% confidence interval = 1.02–2.79, proportion mediated = 64.3%)
Fantozzi et al (2020)	Retrospective cohort study Modified survey from (NHANES) 2013–2014 for taste and smell disorders and the Fox Questionnaire for dry mouth	n = 111 M = 58 (52.3%) F = 53 (47.7%)	57 (48-67)	SARS-CoV-2 positive patients with real-time polymerase chain reaction (RT-PCR) = 111	Xerostomia = 51 (45.9%) Dysgeusia = 66 (59.5%)	Xerostomia, gustatory and olfactory dysfunctions may present as a prodromal or as the sole manifestation of COVID-19
Freni et al (2020)	Cross-sectional study Questionnaire of Olfactory Disorders-Negative Statements (sQOD-NS) The Summated Xerostomia Inventory-Dutch Version (SXI-DV) The Standardised Patient Evaluation of Eye Dryness (SPEED) Schirmer test I The Hearing Handicap Inventory For Adults (HHIA) Tinnitus Handicap Inventory (THI)	n = 50 M = 30 (60%) F = 20 (40%)	37.7 ± 17.9	Laboratory-confirmed COVID-19 infection with real-time polymerase chain reaction (RT-PCR) = 50	During convalescence (Condition A): Gustatory dysfunction = 35 Xerostomia = 16 After 15 days from RT-PCR SARS-COV-2 negativity (Condition B): Gustatory dysfunction = 4 Xerostomia = 1	During convalescence (Condition A): OR (95% CI) - Gustatory dysfunction = 26.81 (8.1–87.9) - Xerostomia = 23.05 (2.9-182.2)
Biadsee et al (2020)	Case series Questionnaire	n = 128 M = 58 F = 70	36.25 (18-73)	SARS-CoV-2 positive patients with real-time polymerase chain reaction (RT-PCR), ambulatory, nonhospitalised patients	Taste disorders = 67 Dry mouth = 72 Facial pain = 18 (women) Masticatory muscle pain = 15 Additional oral manifestations: - Plaque-like changes in the tongue = 9 - Swelling = 10 - Oral bleeding = 6	A considerable number of patients presented with olfactory and oral disorders. Impaired sense of taste was observed in 52% of the patients and xerostomia in 56%. Anosmia and facial pain were more common among women (p < 0.001 and p = 0.01, respectively)
Politi et al (2020)	Retrospective analysis	n = 111 (total number of patients seen by the Oral and maxillofacial surgery specialists during the initial six weeks of COVID-19 lockdown in 2020) M = 64% F = 36%	42 (6-72)	NR: Patients with cervicofacial infection of dental etiology referred to maxillofacial surgery during the initial six weeks of COVID-19 lockdown in 2020 compared with the equivalent period in the two preceding years = 22	Cervicofacial infection during COVID-19 lockdown in 2020 = 22 - Patients requiring hospital admission = 10 (45%) - Post extraction infections = 0 (0%) - Total patients receiving antibiotics = 22 (100%) - Oral = 7 (32%) - IV Single dose + Oral = 5 (23%) - IV Inpatient = 10 (45%) - Patients having antibiotic therapy prior to presentation to OMFS = 12 (55%) - Admitted patients undergoing incision and drainage (extraoral / intraoral) = 10 (100%) - General anaesthesia = 8 (80%) - Local anaesthesia = 2 (20%) - Extraoral drainage = 5 (50%) - Intraoral drainage = 5 (50%) - Not admitted patients = 12 (55%) - Incision and drainage under local anaesthesia = 7 (58%) - No treatment = 5 (42%)	During COVID-19 lockdown in 2020: - Overall decrease in the number of cases seen with cervicofacial infection of dental origin - A high percentage of those seen required hospital admission - They required more invasive treatment with an increase in intraoral drainage under LA and extraoral drainage under GA - There was a decrease in the length of in-patient stay
Wu et al (2021)	Retrospective analysis	n = 4158 Pre-SARS-COV-2 = 1716 M = 873 (50.9%) F = 843 (49.1%) SARS-COV-2 = 2442 M = 1236 (50.6%) F = 1206 (49.4%)	Pre-SARS-COV-2 = 24.7 ± 16.7 SARS-COV-2 = 33.0 ± 19.4	NR: Dental emergency patients in the SARS-COV-2 period (January 20 to March 8, 2020), compared with the number of patients admitted to the emergency center before the SARS-COV-2 pandemic (January 21 to March 10, 2019)	Acute pulpitis and/or acute apical periodontitis: - Pre-SARS-COV-2 = 413 (25.9%) - SARS-COV-2 = 858 (35.1%) Acute gingivitis and/or acute pericoronitis: - Pre-SARS-COV-2 = 314 (18.2%) - SARS-COV-2 = 529 (21.7%) Temporomandibular joint disorders: - Pre-SARS-COV-2 = 24 (1.4%) - SARS-COV-2 = 56 (2.3%) Cellulitis and abscess of the oral cavity: - Pre -SARS-COV-2 = 161 (9.6%) - SARS-COV-2 = 227 (9.3%) Open wound of the lip and oral cavity: - Pre -SARS-COV-2 = 695 (38.0%) - SARS-COV-2 = 492 (20.1%) Fracture of tooth: - Pre-SARS-COV-2 = 58 (3.2%) - SARS-COV-2 = 23 (0.9%) Others (prosthesis, aesthetic, recall, or maintenance): - Pre-SARS-COV-2 = 51 (3.7%) - SARS-COV-2 = 257 (10.5%)	During the SARS-COV-2 pandemic, the number of dental emergency visits increased by 29.7%. Trauma, acute pulpitis, and acute periodontitis were the leading causes of patients visiting the dental emergency
Ramírez et al (2021)	Observational study	n = 261 M = 128 F = 133	20 months to 87 years	NR: Odontostomatological emergencies (OSE) treated between March 17th and 4th of May 2020 in four dental clinics in Madrid (Spain)	Acute apical periodontitis = 143 (54.7%) Acute irreversible pulpitis = 32 (12.2%) Dental fracture = 25 (9.5%) Pericoronaritis = 17 (6.5%) Odontogenic abscess = 15 (2.3%) Prosthetics = 29 (11.1%)	The most prevalent pathology was acute apical periodontitis, whereas odontogenic abscess showed the lowest frequency. Prosthetic-orthodontic OSE represented 14% of cases
Sirin et al. (2021)	Retrospective analysis	n = 137 M = 71 (52%) F = 66 (48%)	20–65	Positive real time PCR COVID-19 test	Dental damage stage 0 = 31 Chronic disease = 1 (3%) Number of dental caries = 0.2 ± 0.4 Number of missing teeth = 3.9 ± 3.2 Number of hospitalisations due to COVID-19 = 0 (0%) Symptom associated with COVID-19 = 11 (35%) - Dental damage stage 1 = 30 Chronic disease = 3 (10%) Number of dental caries = 1.0 ± 1.2 Number of missing teeth = 5.2 ± 5.7 Number of hospitalisations due to COVID-19 = 3 (10%) Symptom associated with COVID-19 = 25 (83%) Dental damage stage 2 = 44 Chronic disease = 20 (45%) Number of dental caries = 1.9 ± 1.5 Number of missing teeth = 6.4 ± 5.6 Number of hospitalisations due to COVID-19 = 13 (30%) Symptom associated with COVID-19 = 39 (89%) Dental damage stage 3 = 32 Chronic cisease = 17 (53%) Number of dental caries = 2.5 ± 2.0 Number of missing teeth = 10.0 ± 6.5 Number of hospitalisations due to COVID-19 = 24 (75%) Symptom associated with COVID-19 = 31 (97%)	Dental damage stage: - Positive high correlation with number dental caries (NDC) - Positive moderate correlation with number of hospitalisations due to COVID-19 (NHC) and Symptom associated with COVID-19 (SAC) - Positive moderate correlation with presence of chronic disease (CD) - Dental damage stage 3 : Significantly higher age and mortality - Chronic disease (CD), numbers of dental caries (NDC) and hospitalisation due to COVID-19 (NHC) values were higher in dental damage stage 2 and 3 than in dental damage stage 0 and 1. - Hospitalisation due to COVID-19 (NHC) were higher in dental damage stage 3 than in dental damage stage 2 - Missing teeth (NMT) were higher in dental damage stage 3 than other stages. - Symptom associated with COVID-19 (SAC) were significantly lower in dental damage stage 0 than in dental damage stage 1, 2 and 3 - Numbers of dental caries (NDC), hospitalisation due to COVID-19 (NHC), Symptom associated with COVID-19 (SAC) and Chronic disease (CD) were effective on DD staging; they were moderately positively related.
Kamel et al (2021)	Cross-sectional questionnaire survey	n = 308 M = 104 (33.8%) F = 204 (66.2%)	34.98 ± 8.08	SARS-CoV-2 confirmed by (RT-PCR) Severe COVID-19 description: - High respiratory rate (>30 breaths/min) - Heart rate >100 beats/min - Severe dyspnoea or chest pain - Oxygen saturation <93% - High-grade fever (>39 °C) - Hospitalised patients who required oxygen or intensive care unit admission	Poor oral health: - Severe COVID-19 = 52 (65%) - Mild COVID-19 = 12 (5.3%) 6 weeks recovery period n = 26 (40.6%) 4 weeks recovery period n = 28 (43.8%) 2 weeks recovery period n = 10 (15.6%) Fair oral health: - Severe COVID-19 = 20 (25%) - Mild COVID-19 = 146 (64%) 6 weeks recovery period n = 18 (10.8%) 4 weeks recovery period n = 54 (32.5%) 2 weeks recovery period n = 94 (56.6%) Good oral health: - Severe COVID-19 = 8 (10%) - Mild COVID-19 = 70 (30.7%) 6 weeks recovery period n = 6 (7.7%) 4 weeks recovery period n = 8 (10.3%) 2 weeks recovery period n = 64 (82.1%)	Oral health and COVID-19 severity showed a significant inverse correlation (p<0.001, r = -0.512) Oral health with recovery period and CRP values showed significant inverse correlation (p<0.001, -0.449 and p<0.001, -0.190, respectively)
Salehi et al (2020)	Cross-sectional study	n = 53 M = 23 (43.4%) F = 30 (56.6%)	<50 = 11 (20.7%) ≥50 = 42 (79.3%)	SARS-CoV-2 positive patients	Oropharyngeal candidiasis (OPC) = 53 Time interval between diagnosis of COVID-19 and clinical presentations of OPC = 8 days (range: 1-30 days) - Principal underlying conditions: CV diseases = 28 (52.8%) Diabetes = 20 (37.7%) - The most common risk factor: Lymphopaenia = 38 (71.7%) Recipient broad-spectrum antibiotics = 49 (92%) Corticosteroid therapy = 25 (47%) Admission to ICU = 26 (49%) Mechanical ventilation = 16 (30%) - Distribution of *Candida* species: 65 *Candida* isolates causing OPC *C. albicans* (70.7%) *C. glabrata* (10.7%) *C. dubliniensis* (9.2%) *C. parapsilosis* sensu stricto (4.6%) *C. tropicalis* (3%) *C. krusei* (1.5%)	Concerns regarding the occurrence of OPC in Iranian COVID-19 patients
Hocková et al (2021)	Case series	n = 9 Gender NR for all the patients, only for oral complications patients: M = 3 F = 0	NR for all the patients, only for oral complications patients: N 1 = 68 N 2 = 61 N 3 = 64	SARS-CoV-2 positive critically ill patients (RT-PCR) admitted to the intensive care units (ICUs)	Oral complications = 3 (33. 3%) - Haemorrhagic ulcers and necrotic ulcers affecting the lips and tongue - Presence of opportunistic pathogens, confirming the possibility of co-infection	Three out of nine critically ill patients (33.3%) presented with oral complications including haemorrhagic ulcers and necrotic ulcers affecting the lips and tongue
Sinjari et al (2020)	Observational study Questionnaire of 32 questions	n = 20 M = 55% F = 45%	69.2 (35-91)	Hospitalised patients for COVID-19	Oral manifestation of the hospitalised patients for COVID-19: - Xerostomia, none of the patients reported xerostomia before contracting the virus, whilst during hospitalisation the percentage increased to 30% (p = 0.02) - Impaired taste = 25% of patients during hospitalisation - Burning sensation = 15% of patients during hospitalisation - Difficulty in swallowing = 20% of patients during hospitalisation	Importance of the close link between SARS-CoV-2 and oral manifestations. There is no scientific evidence in the literature that certifies which oral symptoms SARS-CoV-2 can cause
Favia et al (2021)	Case-Series	n = 123 M = 70 F = 53	Form of Covid-19 - Moderate: 63 - Severe: 74 - Critical: 81	SARS-CoV-2 (confirmed by RT-PCR) hospitalised patients	Form of COVID-19: Moderate: n = 95 (77%) Type of oral lesions: - Geographic tongue = 5 - Fissured tongue = 4 - Ulcerative lesion = 51 - Blisters = 14 - Hyperplasia of papillae = 33 - Angina bullosa = 8 - Candidiasis = 18 - Ulcero-necrotic gingivitis = 1 - Petechiae = 4 Oral Symptoms: - Pain - Burning - Bleeding - Difficulty to chewing and swallow - Taste disorders = 87% Severe: n = 21 (17%) Type of oral lesions: - Geographic tongue = 2 - Fissured tongue = 1 - Ulcerative lesion = 11 - Blisters = 5 - Hyperplasia of papillae = 13 - Angina bullosa = 2 - Candidiasis = 4 - Ulcero-necrotic gingivitis = 2 - Petechiae = 6 Oral symptoms: - Pain - Burning - Bleeding - Difficulty to chewing and swallow - Taste disorders = 88% Critical: n = 8 (6%) Type of oral lesions: - Ulcerative lesion = 3 - Hyperplasia of papillae = 2 - Angina bullosa = 1 - Candidiasis = 6 - Ulcero-necrotic gingivitis = 4 - Petechiae = 4 - Spontaneous oral hemorrhage = 1 Oral symptoms: - Pain - Burning - Bleeding - Difficulty to chewing and swallow - Taste disorders = 83%	Oral lesions in 65.9% occurred in the early stage of Covid-19 before the beginning of specific therapies. Moreover, this study discovered that the physio-pathological mechanism underlying the formation of early oral lesions is the thrombosis of sub-epithelial and deeper vessels. The presence of oral lesions in early stages of Covid-19 could represent an initial sign of peripheral thrombosis, a warning sign of possible development to severe illness. This suggests that anticoagulant therapies should start as soon as possible
Huang et al (2021)	Prospective cohort study	9 samples of human minor salivary glands and gingiva (13,824 cells) NR	NR	SARS-CoV-2 positive patients	They confirmed SARS-CoV-2 infection in the glands and mucosae. Saliva from SARS-CoV-2-infected individuals harbored epithelial cells exhibiting ACE2 and TMPRSS expression and sustained SARS-CoV-2 infection	Acellular and cellular salivary fractions from asymptomatic individuals were found to transmit SARS-CoV-2 ex vivo. Matched nasopharyngeal and saliva samples displayed distinct viral shedding dynamics, and salivary viral burden correlated with COVID-19 symptoms, including taste loss. Upon recovery, this asymptomatic cohort exhibited sustained salivary IgG antibodies against SARS-CoV-2
Jimenez-Cauhe et al (2020)	Case-Series	n = 21 Gender NR for all the patients, only for Enanthem patients (6 patients) M = 2 (34%) F = 4 (66%)	NR for all the patients, only for Enanthem patients (6 patients) 40 - 69 years	SARS-CoV-2 positive patients	Enanthems located in palate = 6 Petechial = 2 Macular with petechiae = 3 Macular = 1	Presence of enanthem is a strong clue that suggests a viral etiology rather than a drug reaction, especially when a petechial pattern is observed
Nuño González et al (2021)	Cross-sectional study	n = 666 NR	NR	Hospitalised patients with coronavirus disease 2019 pneumonia	Oral mucosal changes = 78 (11,7%) - Transient U-shaped lingual papillitis = 35 (11.5%) - Tongue swelling = 20 (6.6%) - Glossitis with patchy depapillation = 12 (3.9%) - Mucositis = 12 (3.9%) - Aphthous ulcers = 21 (6.9%) - Burning mouth = 16 (5.3%) - White tongue = 5 (1.6%) - Candidiasis = 3 (1%) - Enanthema = 2 (0.5%)	COVID-19 also manifests in the oral cavity. The most common manifestations are transient U-shaped lingual papillitis, glossitis with patchy depapillation, and burning mouth syndrome
Fidan et al (2021)	Prospective study	n = 74 M = 49 (66.2%) F = 25 (33.8%)	51.4 ± 6.3	SARS-CoV-2 confirmed by (RT-PCR) patients	Oral lesions and distribution oral lesion areas = 58 - Aphthous-like ulcer = 27 - Erythema = 19 - Lichen planus = 12 The most common location of lesions - Tongue = 23 - Buccal mucosa = 20 - Gingiva = 11 - Palate = 4	Oral lesions in fifty-eight of seventy-four Covid 19 patients. There are limited reports about oral lesions in patients with Covid 19
AbuBakr et al (2021)	Cross-sectional online questionnaire survey	n = 573 M = 165 F = 408	36.19 ±9.11 (19–50)	SARS-CoV-2 confirmed by (RT-PCR): – Mild-to-moderate symptoms, without severe respiratory failure, not hospitalised	Occurrence of oral manifestations = 411 (71.7%) Pain in jaw bones or joint = 69 (12%) Halitosis = 60 (10.5%) Ulcerations = 117 (20.4%) Xerostomia = 273 (47.6%) Oral or dental pain = 132 (23%) Combined manifestations = 162 (28.3%)	Mild-to-moderate cases of COVID-19 infection are associated with oral symptoms. Statistically significant difference in the incidence of oral manifestations in relation to the oral hygiene measures taken by the patients
Subramaniam et al (2021)	Observational study	n = 713 M = 416 F = 297	NR for all the patients, only for patients with oral lesions (9 patients) 43-70 years	SARS-CoV-2 confirmed by (RT-PCR)	Differential diagnosis: - Patient 1 = Traumatic ulcer secondary to cheek bite - Patient 2 = Diabetic mucositis - Patient 3 = Geographic tongue (related to psychosomatic disorders) and traumatic ulcers - Patient 4 = Acute/chronic pseudomembranous candidiasis (thrush) - Patient 5 = Geographic tongue and nutritional deficiency mucositis - Patient 6 = recurrent herpetic labialis. Nutritional deficiency angular cheilitis - Patient 7 = traumatic ulcer with bloody encrustations (history of intubation) - Patient 8 = ulceration secondary to nutritional deficiency/benign migratory glossitis - Patient 9 = mucositis secondary to anemia and xerostomia	Among 713 patients positive to coronavirus, who were screened for oral lesions, only nine patients had complaints. No specific pattern or characteristic oral lesions were noted in a study of 713 COVID-positive patients to qualify these lesions as oral manifestations of SARS-CoV-2 infection
Emodi-Perlman et al (2020)	Cross-sectional online survey (regarding TMD, bruxism, anxiety and depression: 3Q/TMD, possible/probable bruxism, and Patient Health Questionnaie-4, as detailed below)	n = 1792 From Israel = 700 M = 235 (33.6%) F = 465 (66.4%) From Poland = 1092 M = 454 (41.6%) F = 638 (58.4%)	From Israel = 700 18–35 = 203 36–55 = 283 >56 = 200 N/A = 14 From Poland = 1092 18–35 = 828 36–55 = 234 > 56 = 30 N/A = 0	NR Subjects selected from two culturally different countries during the lockdown: Israel and Poland	TMD Positive: - Israel = 152 - Poland = 576 TMD Negative: - Israel = 548 - Poland = 516 Awake bruxism (AB): - Israel: Probable AB (I) = 153 Possible AB (II) = 125 AB Negative (III) = 422 - Poland: Probable AB (I) = 378 Possible AB (II) = 365 AB Negative (III) = 349 Sleep bruxism (SB): - Israel: Probable SB (I) = 152 Possible SB (II) = 70 SB Negative (III) = 478 - Poland: Probable SB (I) = 351 Possible SB (II) = 199 SB Negative (III) = 542	TMD: Odds of occurrence of TMDs among the Polish young adult and adult age groups (18–35 years and 36–55 years) were significantly higher for both males and females as compared to the Israeli groups (odds ratios ranged from 3.04 to 5.37). Awake bruxism (AB): Odds of occurrence among the Polish participants were significantly higher in general than among the Israeli participants (except the young and elder males), with the odds ratios ranging between 2.51 and 6.41. Sleep bruxism (SB): Odds of occurrence among the Polish subjects (except for males in the two higher age groups) were similar to those of the Israeli subjects, with the odds ratios ranging from 1.4 to 3.99
Asquini et al (2021)	Prospective cohort study	n = 40 Chronic TMD = 16 - M = 6% - F = 94% Acute/sub- acute TMD = 24 - M = 12% - F = 88%	Chronic TMD = 28 (median) Acute/sub- acute TMD = 29 (median)	NR Patients with one or multiple TMD diagnoses before the Covid-19 outbreak and followed-up performed immediately after the lockdown period	Chronic TMD = 16 Acute/sub-acute TMD = 24	COVID Stress Scales (CSS) were significantly higher in those with chronic TMDs compared to those with acute/subacute TMDs (p<0.05)
Petrescu et al (2020)	Retrospective Cross-sectional study	April 2020 n = 724 M = 53.59% F = 46.41%	April 2020 = 2-96	NR Patients seeking emergency dental services at the Emergency Department of County General Hospital and “Iuliu Hat, ieganu” University of Medicine and Pharmacy, Cluj-Napoca, Romania, in April 2020	Disease type and diagnosis: April 2020 = 724 Acute pulpitis = 244 Acute apical periodontitis = 324 Pericoronitis of the impacted teeth = 11 Post-extractional alveolitis = 1 Abscess = 73 Dislocation temporo-mandibular joint = 1 Dento-alveolar trauma = 40 Ulcer-necrotic gingivitis = 6 Orthodontic appliance irritation injury = 7 Prosthetic crown loosening = 12 Pathological dental mobility = 12 Other injury (irritative, ulcerative, decubitus) = 43 Caries = 13	Treatment of oral emergencies: April 2020: Sedative filling = 191 (29.28%) Drainage (endodontic) = 92 (14.09%) Drainage/antiseptic lavage = 75 (11.46%) Drainage/sedative filling = 6 (0.97%) Extraction = 14 (2.07%) Incision/drainage = 8 (1.24%) Incision/drainage/antiseptic lavage = 3 (0.55%) Antiseptic lavage = 12 (1.8%) Pulpectomy = 11 (1.66%) Suppression of irritant factor = 12 (1.8%) Filling = 0 (0%) Other = 31 (4.7%) Examination/consultation only = 198 (30.36%)
Zhang et al (2021)	Nationwide online cross-sectional survey of 22 questions (24 January 2020 to 2 May 2020)	n = 3352 M = 1217 (36.31%) F = 2135 (63.69%)	18-30 = 1241 (37.02%) 31-50 = 1582 (47.19%) ≥51 = 529 (15.79%)	NR Participants from Wuhan and other places of China (24 January 2020 to 2 May 2020)	Oral problems: - Wuhan = 63.30% - Other places of China = 57.07% Global oral problems: - Gingival bleeding = 23% - Bad breath = 20% - Oral ulcers = 17% - Other problems = 14% - Swelling = 12% - Toothache = 9% - TMJ disorders = 5%	Significantly more participants in Wuhan (63.30%) experienced oral problems than other places in China (57.07%). Gingival bleeding, bad breath and oral ulcers were the three most common oral problems amid the epidemic. Toothbrushing frequency did not differ significantly between participants from Wuhan and other places and was associated with the prevalence of oral problems

### Study Outcomes

#### COVID-19 and periodontitis

Most of the included studies demonstrate an association between periodontal diseases and COVID-19. In a case-control study, severe periodontitis (OR = 11.75; 95% CI, 3.89 to 35.49), plaque score (OR, 7.01; 95% CI, 1.83 to 26.94) and gingival bleeding (OR, 17.65; 95% CI, 5.95 to 52.37) were more frequently observed in COVID-19 patients,^4^ whereas another such study failed to find it.^33^ The use of self-reported indicators to define a periodontal case, such as painful or bleeding gums, may have biased the results.^33^ The periodontal status of 82 positive SARS-CoV-2 patients was assessed, according to the new classification of periodontal diseases.^55^ Patients diagnosed with stages II-IV of periodontitis had increased odds of requiring assisted ventilation (OR = 7.45, 95% CI 2.00–25.82), hospital admission (OR = 36.52, 95% CI 4.62–288.64), death (OR = 14.58, 95% CI .69–125.33) or developing COVID-19 pneumonia (OR = 4.42, 95% CI 1.57–12.45).^22^ In a case-control study with 568 positive patients, it was observed that periodontal patients had an increased risk of COVID-19 complications (OR = 3.67, 95% CI 1.46–9.27), death (OR = 8.81, 95% CI 1.00–77.7), intensive care unit (ICU) admission (OR = 3.54, 95% CI 1.39–9.05) and need for assisted ventilation (OR = 4.57, 95% CI 1.19–17.4).^36^ During the lockdown period, the risk of contracting COVID-19 had a direct effect on reducing dental visits. Patients who skipped their regular visits had a higher OR for periodontitis (OR, 1.68; 95% CI, 1.02-2.79).^28^ At the histological level, the presence of and possible tissue alterations due to SARS-CoV-2 in the periodontium were investigated by minimally invasive post-mortem biopsies in 7 fatal cases of COVID-19.^37^ Among the 7 collected samples, 5 were positive for SARS-CoV-2, and the presence of the virus was correlated with morphologic alterations of the keratinocytes forming the junctional epithelium.

#### COVID-19 and xerostomia

The association between COVID-19 and xerostomia was evaluated in a retrospective cohort study,^15^ including 111 COVID-19 patients. Xerostomia was evaluated with the Fox questionnaire. Among COVID-19 patients, 51 reported xerostomia, with a median dryness score of 5/11. Of those, 10 also reported xerostomia as a prodromal symptom of SARS-CoV-2 infection with a median onset time of 7 days before COVID-19 diagnosis. Swallowing differences were reported in 20 patients with dry-mouth symptoms, with 14 experiencing difficulties only with dry foods and 19 requiring the use of adjuvant liquids during the swallowing process. In this study, 66 patients also reported taste disorders and 46 olfactory disorders. Similarly, 16 out of 50 positive SARS-CoV-2 patients reported mouth dryness after completing the Summated Xerostomia Inventory-Dutch Version (SXI-DV), indicating xerostomia as an early symptom of COVID-19.^18^ In a case series, 140 COVID-19 patients answered an online questionnaire regarding early manifestations such as xerostomia, olfactory and taste functions. Dry mouth was reported by 56% of patients, indicating that this symptom might be considered in early detection and diagnosis.^8^

#### COVID-19 and candidiasis

According to Salehi et al,^46^ COVID-19 patients have a high risk of mucosal candidiasis, with *Candida albicans* being the most common causal yeast (70.7% of patients). In this study, cardiovascular illnesses (52.83%) and diabetes (37.7%) were the most common underlying diagnoses among the 53 COVID-19 patients with oropharyngeal candidiasis; lymphopaenia was the most common recognised risk factor (71%). Systemic conditions such as a compromised immune system and long-term pharmacotherapies may worsen oral conditions in SARS-CoV-2 infected patients.

#### COVID-19, temporomandibular disorders and bruxism

Two concomitant cross-sectional online surveys evaluated the impact of COVID-19 lockdown on the prevalence and worsening of temporomandibular disorders (TMDs) and bruxism symptoms.^14^ In two different countries (Israel and Poland), anxiety, depression, or personal concerns significantly increased the odds of the occurrence and aggravation of TMDs. Among the Polish population, 52.7% experienced clinical symptoms of TMDs, 34.6% experienced waking bruxism and 32% sleep bruxism; such manifestations were seen in 21.7%, 21.8%, and 21.7% of Israeli subjects, respectively. Demographic factors and the social context of both countries could explain the significant differences in the results. A retrospective cohort study aimed to evaluate the role of COVID-19 lockdown in Italian patients with TMD. Different tests, such as the Hospital Anxiety and Depression Scale (HADS) and COVID Stress Scale, multiple variables such as age and gender, and pain characteristics such as intensity and disability, were considered throughout the study. The results showed that COVID-19 distress negatively influenced the anxiety and depression levels of patients with chronic TMD, increasing facial pain severity.^5^

#### COVID-19 and oral mucosal lesions

Oral lesions could also be a complication during SARS-CoV-2 infection. Favia et al^16^ reported different types of oral lesions in COVID-19 patients with regard to time of onset and the beginning of COVID-19–specific treatments. Most of the oral lesions occurred alongside the systemic symptoms, and they appeared before viral treatment with antibiotic combined therapies, corticosteroids, and anticoagulants, suggesting oral examination as a potential tool for early diagnosis.^16^ In a retrospective^17^ and a cross-sectional study,^20^ 58 out of 74 (78%) and 78 out of 666 (11.7%) SARS-CoV-2 patients presented oral manifestations, respectively. One of the most frequent changes detected were U-shaped lingual papillitis (11.5%),^20^ and apthous-like ulcers (46.6%).^17^ Another study evaluated the presence of enanthem.^29^ Out of 21 patients with COVID-19 and skin rash, 6 presented an enanthem that was either macular, petechial, or macular with petechial.^29^ Twenty hospitalised patients with COVID-19 answered a questionnaire about their oral health condition. A decrease in the quality of oral hygiene and an increase in symptoms such as xerostomia, burning sensation, impaired taste and difficulty swallowing were noted during coronavirus infection.^50^ Another questionnaire was answered by 573 Egyptian patients who were characterised as mild-to-moderate cases of COVID-19, 71.7% of whom reported an oral complication, with xerostomia (47.6%) being the most frequent manifestation, followed by oral or dental pain (23%) and ulcerations (20.4%).^1^ These two surveys highlight the importance of oral examination of such patients. During the admission to ICUs, 3 out of 9 patients presented oral complications including haemorrhagic and necrotic ulcers which were related to prolonged ventilatory support.^23^ An observational study investigated the hypothesis that oral ulcers are present due to COVID-19 infection. Out of 713 positive patients, only 9 reported mouth discomfort (1.26%) such as geographic tongue, angular cheilitis and ulcers. The authors suggested that these lesions might be due to either concomitant systemic diseases, local irritants or co-existing lesions.^54^ An adult human oral scRNA-seq atlas^25^ was generated to explore the role of the oral cavity in COVID-19 transmission. RNA and protein expression assessments from human minor salivary glands and gingiva were collected and analysed from nine samples. Infection with the virus was confirmed in both glands and mucosae. The expression of ACE2 and TMPRSS receptors with detection of SARS-CoV-2 infection was observed in the saliva of COVID-19 patients. Salivary fractions of asymptomatic patients were also a potential route of COVID transmission, indicating that the oral cavity may play an important role in the virus’s dissemination.^25^

#### COVID-19 and oral health

Oral and general health are strongly associated. Retrospective and cross-sectional studies explored the potential link between the oral health status and the severity of COVID-19 disease. Sirin et al^51^ included full oral and panoramic radiographic examination of 137 patients and observed a significant correlation between poor oral health status and the morbidity and mortality of this viral disease. Similarly, Kamel et al^30^ questioned 308 SARS-CoV-2–positive Egyptians and suggested that patients with poorer oral health had a more severe form of COVID-19 and a delayed recovery period. The impact of COVID-19 lockdown in dental emergencies was investigated in different countries. An increase of 77.9% and 29.7% of patients seeking emergency dental treatment was observed in Romania^41^ and China,^60^ respectively, between 2019 and 2020. In contrast, a reduction of 76.3% and 93% was noted in the United Kingdom^42^ and India,^32^ respectively, comparing the pre-lockdown and lockdown periods. Politi et al^42^ also recorded an increase in patients requiring admission to the hospital (45%), compared with 37% in 2018 and 31% in 2019. It can be assumed that due to the lockdown, people would not start seeking emergency dental services until a highly acute situation occurred.^42^ The main causes of seeking expert help were acute apical periodontitis, abscess, and acute pulpitis. Acute apical periodontitis ranged from 35.1%^60^ to 54.7%,^43^ followed by oral abscesses, ranging from 16.6%^32^ to only 2.3%.^43^ An online cross-sectional questionnaire survey assessed the impact of the COVID-19 pandemic on the oral health of adults in Wuhan and China.^62^ Oral problems were experienced by 59.9% of the participants, with 63% living in Wuhan. During the pandemic, the most commonly detected oral symptoms were gingival bleeding (23%), bad breath (20%), and oral ulcers (17%), which were associated with worse oral health.^62^

## Discussion

COVID-19 is at the center of attention of both the scientific community and the general population. In March 2020, the WHO declared COVID-19 a pandemic and, concomitantly, an increase in the number of publications was observed. Regarding the oral cavity, different signs of SARS-CoV-2 infection have been highlighted, including taste loss, xerostomia, and oral lesions, which may facilitate early disease diagnosis.

Different functions, including speaking, coughing, and sneezing, are involved in virus transmission.^53^ To infect the tissues and organs, SARS-CoV-2 displays mechanisms to invade different cell types, and its entry occurs mainly through the ACE2 and TMPRSS2 receptors.^24^ Therefore, cells that bear such receptors are susceptible to infection by SARS-CoV-2.^64^ Several studies have demonstrated the presence of ACE2 and TMPRSS2 receptors in oral tissues.^45^ In 2020, Xu et al^61^ reported a high expression of the ACE2 receptor in epithelial cells of the tongue. However, ACE2 and TMPRSS2 are also expressed in the salivary glands, potentially explaining one of the viral transmission mechanisms through saliva.^52^ In line with earlier research, Huang et al^25^ confirmed the expression of ACE2 and TMPRSS2 receptors in minor salivary glands and mucosa. The presence of SARS-CoV-2 in periodontal tissues was also confirmed in post-mortem biopsies in 5 of 7 fatal cases of COVID-19.^37^ Those findings confirmed that the oral cavity and oral tissues are potential virus reservoirs.

This review aimed to describe the most common signs and symptoms of SARS-CoV-2 in the oral cavity. A large number of articles have been published, listing all the changes detected in the oral cavity during infection by this virus. However, most of these studies are case reports or letters to editors, and were not included in this review. Based on a review carried out in 2021,^27^ the most common oral features of COVID-19 include aphthous-like lesions, herpetiform lesions, candidiasis, and oral lesions of Kawasaki-like disease. In that study, the patient’s age and the severity of COVID-19 illness were the most common factors that predicted oral lesions.^27^ The significance of oral lesions as a possible early sign of severe COVID-19 cases has already been emphasised. Nevertheless, it is meaningful to critically appraise these conclusions, as oral changes might be due to either concomitant systemic diseases, pre-existing lesions, or local irritants.^54^

A better knowledge of possible risk factors associated with severe COVID-19 illness is critical. Elderly age, male gender, diabetes, obesity, and hypertension are some examples.^19^ Indeed, there is a higher frequency of obese and elderly patients admitted to ICUs for COVID-19.^19,48^ Another risk factor is smoking. Smokers seem to have 3.25-times greater odds of developing a severe form of COVID-19 in comparison with non-smokers.^21^ As these parameters are well-described risk factors for oral diseases, notably periodontal diseases, it is of paramount importance to conduct clinical trials comparing COVID-19 patients and matched controls.

Quarantine, social distancing, or total lockdown are demonstrated to have a psychological, social, and economic impact on the general population,^39^ which may indirectly affect oral health.^14^ A recent systematic review and meta-analysis^12^ estimated the mental health consequences of the COVID-19 pandemic. During the coronavirus pandemic, the rates of depression, anxiety, imsommia, and psychological distress reached a prevalence of 15.97%, 15.15%, 23.87%, and 13.29%, respectively.^12^ Asquini et al^5^ demonstrated an increased susceptibility to COVID-19 distress in patients with chronic TMDs which could amplify anxiety, depression, and chronic facial pain.

It is important to mention that periodontal diseases and COVID-19 seem to share risk factors, such as age, obesity, diabetes, smoking and stress. Notably, periodontal disease has been linked with diabetes, metabolic syndrome, obesity, eating disorders, liver disease, cardiovascular disease, Alzheimer’s disease, rheumatoid arthritis, unfavorable pregnancy outcomes, and cancer.^31^ These factors may increase the likelihood of an association between periodontitis and SARS-CoV-2 infection.

COVID-19 restrictions also had a negative impact on patients’ oral health care needs.^47^ The COVID-19 lockdown resulted in the postponement of many non-emergency or highly infectious dental procedures. In Wuhan, an online questionnaire survey showed a deterioration of oral health in 63.3% of the population during the COVID-19 lockdown. 39.4% of the population stated unavailability of dental services as the reason.^62^ In Taiwan, the utilisation of ambulatory medical and dental visits during COVID-19 was compared with the same period in 2019. The greatest reduction in dental visits was at hospitals, while the reduction in dental clinic visits was lower.^34^ Conversely, a reduction in patients seen with cervicofacial infection of dental aetiology was observed in London, compared with the same period the year before. This could be due to people following government advice to stay at home, as well as the efficient functioning of emergency dental care given by urgent dental-care centers.^42^

Protective protocols for dental care to curtail the spread of SARS-CoV-2 were immediately implemented in offices and clinics across the globe.^7^ Many recommendations and standards for reopening dental clinics have been proposed.^7^ In December 2020, the World Health Organization (WHO) announced biosecurity recommendations for health workers to consider during patient treatment, in an effort to reduce the risk of virus exposure.^57^ In addition, the American Dental Association (ADA) created standards for dental offices to protect patients and dental staff, as well as to limit the danger of COVID-19 transmission.^11^ Some of the protective measures that were recommended are the delay of non-urgent dental care and the treatment of high-risk patients in the early hours of a dental office’s opening. At the time of check-in, patients should be screened by taking their temperature.^7^ All health professionals should utilise droplet and contact precautions (medical masks, gowns, gloves, and eye protection) as needed. Waiting rooms and reception areas should provide adequate ventilation. Hand wash or hydroalcoholic solutions for hand disinfection should be available upon entrance to the dental office.^7^ Compliance with these measures aims to improve patient trust, ensure continuity of dental treatment, and prevent degradation of oral health status during the pandemic. Furthermore, the COVID-19 vaccine seems to have high acceptance among dentists, providing a sense of security to patients.^10^

Among 138 patients hospitalised with COVID-19 in Wuhan, 26% of the patients required intensive care.^56^ Oral complications are commonly observed in patients during their stay in ICU. In a recent study, perioral pressure ulcers, oral candidiasis, herpetic and haemorrhagic oral ulcers, and acute macroglossia were commonly reported complications in critically ill COVID-19 patients.^23^ Long-term prone positioning and mechanical ventilation systems in the ICU, as well as immunosuppressive medications, all contributed to the emergence of oral mucocutaneous problems.^23^ The most common risk factors for oropharyngeal candidiasis, according to Salehi et al,^46^ were the use of broad-spectrum antibiotics and lymphopenia. Patients with a weakened immune system or those on long-term pharmacotherapy were also thought to be at a higher risk of acquiring mucosal candidiasis.^46^ In a recent Cochrane review, frequent toothbrushing and the use of chlorhexidine were found to be effective in preventing ventilator-associated pneumonia in critically ill patients.^63^

This review has some limitations. Most importantly, there is an absence of high-quality studies. There are currently no studies that allow us to draw conclusions about the impact of COVID-19 on oral health, because the majority of them are cross-sectional studies. It is difficult to compare studies due to the heterogeneity of diagnostic methods and criteria. For example, some studies used only questionnaires to diagnose patients with either SARS-CoV-2 infection or oral diseases. The absence of control groups and the great variety of COVID symptoms make it difficult to investigate the actual role of the virus in the oral cavity. To demonstrate the causal relationship between oral lesions and COVID-19, further prospective and longitudinal investigations comparing SARS-CoV-2 negative and positive individuals are needed.

## Conclusion

An effort was made to summarise and examine all the ways that COVID-19 may influence oral health. To improve the oral health of vulnerable hospitalised patients, appropriate professional health training and close communication between healthcare personnel are required.^23^ A better understanding of the mechanisms involved during the SARS-CoV-2 infection will not only be helpful to more precisely determine the involvement of oral tissues in COVID-19 onset and development, but may also open new horizons regarding patient management.
